# Estimation of Muscle Force Based on Neural Drive in a Hemispheric Stroke Survivor

**DOI:** 10.3389/fneur.2018.00187

**Published:** 2018-03-23

**Authors:** Chenyun Dai, Yang Zheng, Xiaogang Hu

**Affiliations:** Joint Department of Biomedical Engineering, University of North Carolina at Chapel Hill and North Carolina State University, Raleigh, NC, United States

**Keywords:** motor unit decomposition, high-density surface electromyogram, neural drive, stroke, neural control, muscle weakness

## Abstract

Robotic assistant-based therapy holds great promise to improve the functional recovery of stroke survivors. Numerous neural-machine interface techniques have been used to decode the intended movement to control robotic systems for rehabilitation therapies. In this case report, we tested the feasibility of estimating finger extensor muscle forces of a stroke survivor, based on the decoded descending neural drive through population motoneuron discharge timings. Motoneuron discharge events were obtained by decomposing high-density surface electromyogram (sEMG) signals of the finger extensor muscle. The neural drive was extracted from the normalized frequency of the composite discharge of the motoneuron pool. The neural-drive-based estimation was also compared with the classic myoelectric-based estimation. Our results showed that the neural-drive-based approach can better predict the force output, quantified by lower estimation errors and higher correlations with the muscle force, compared with the myoelectric-based estimation. Our findings suggest that the neural-drive-based approach can potentially be used as a more robust interface signal for robotic therapies during the stroke rehabilitation.

## Introduction

Stroke survivors manifest impaired hand functions, especially the finger extension of their affected side. The use of robot-assisted devices (e.g., orthosis) (1–3) has shown great promise as a rehabilitation or assistive tool for stroke survivors. In order to control the robot based on user intention, surface electromyogram (sEMG) signals have been widely used as the neural control signal of the assistive robots or orthoses (4–6). However, since the global electromyogram (EMG) can be regarded as a random process at the macro level, directly using EMG as a control input can mask the actual neural control information (7). Moreover, abnormal muscle activation generated from the affected side can lead to less accurate myoelectric control.

In contrast, motoneuron discharge timings have been introduced as a better neural interface to robotic control (7), because the discharge timings can reflect the input signal to the neuromuscular system, and can be more robust for decoding user intent than the traditional EMG-based approach. The motoneuron discharge timing can be extracted from EMG decomposition, and the descending neural drive to the motor unit (MU) pool then can be estimated based on the firing behaviors of the pool. The neural drive can overcome different inconvenient processes in EMG and motor unit action potential (MUAP) that can interfere myoelectric control, such as the crosstalk of multiple EMG channels (9), cancelation of the waveform of MUAPs (8), and variations of MUAPs generated by conductive process from muscle fibers to the skin surface or location shift of electrodes. In addition, the neural-drive-based approach could also overcome the variation or abnormal MUAPs due to the tremor and weakness on the affected side of stroke survivors.

Therefore, we quantified the performance of traditional EMG-based approach and the neural-drive-based approach on estimating the forces of individual finger (index, middle, and ring) extension of a stroke survivor. Our case study provided a promising approach that can help improve the utility of rehabilitation/assistive devices for hand functional recovery of stroke survivors.

## Materials and Methods

### Case Report

We reported a case of an 88-year-old woman who suffered a hemispheric ischemic stroke ten years ago. Her Chedoke assessment scale on the hand section was 3 (moderate impairment). Her primary concern regarding her affected hand was muscular weakness in the hand muscles. The maximin force ratios of the finger extension on the affected side relative to the contralateral side were 0.48, 0.45, and 0.6 for index, middle, and ring fingers, respectively. In addition, the subject had difficulty generating forces with her little finger on her affected side. This study was carried out in accordance with the recommendations of the local Institutional Review Board (IRB) with written informed consent from the subject. The subject gave written informed consent in accordance with the Declaration of Helsinki. The protocol was approved by the local IRB. The written informed consent was obtained from the participant for the publication of this case report.

### Experimental Setup

The subject sat upright in the experimental chair with the tested forearm comfortably placed on a horizontal table and the elbow supported on a foam pad. Her wrist was secured within two padded boards in a neutral position with respect to the flexion/extension to limit the use of her wrist. The four fingers (index, middle, ring, and little) were comfortably abducted. Each finger was secured to one load cell (Interface, SM-200N) for finger force measurement (with 1 kHz sampling rate). During the experiment, the subject was required to isometrically extend one designated finger (index, middle, or ring) each time. First, maximin voluntary contraction (MVC) of each finger was measured when the subject ramped up contraction force until it reached the maximum level and then maintained for 2 s. The average force value during the 2 s contraction plateau was taken as the MVC. After practice trials, she performed different tracking tasks. The subject was asked to track a targeted force trajectory shown on the screen by adjusting the muscle force of a designated finger. Two target trajectories were tested separately such as sine wave and trapezoid. Two contraction levels (20% or 50% MVC) were tested in each target. For the sine-wave target, the force oscillated either from 10% to 20% or from 25% to 50% at the designated force levels. Two repeated trials were performed for each condition. Additionally, the subject was instructed to only extend one of the designated fingers in each trial, but was allowed to activate other fingers, when she has difficulty isolating other fingers. The order of the finger and the contraction level was randomized during the experiment. A 3 min rest was provided between trials to avoid fatigue. The two sides were tested on two separate sessions separated by a week apart. A total of 24 trials (three fingers × two contraction levels × two tracking tasks × two repetitions) were recorded for each side. The force feedback of the designated finger was displayed via a custom-built program using Matlab (MathWorks, Inc.).

**Figure 1:**
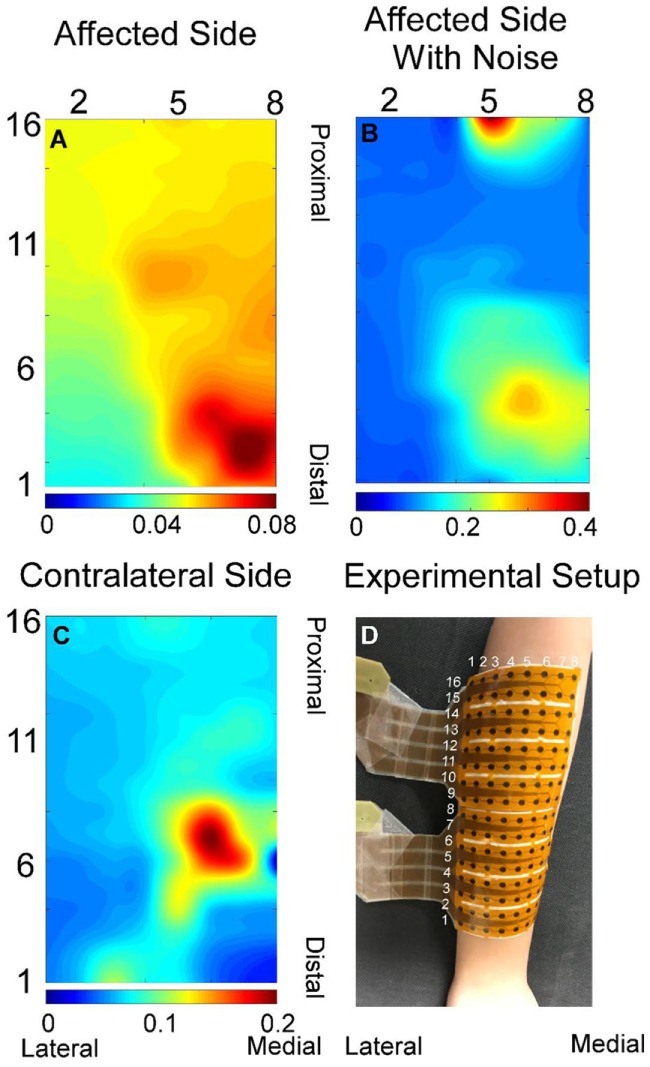
Exemplar root-mean-square map for index finger from both affected **(A)** and contralateral sides **(C)**. **(B)** Root-mean-square map of panel **(A)** with background noise and motion artifact without filtering. **(D)** Experiment electrode placement. All root-mean-square maps were calculated from the trapezoid 50% contraction. All the *X* and *Y* axis labels indicate the row or column number of the electrodes.

Surface EMG signals were recorded over the extensor digitorum communis muscle (EDC) using an 8 × 16-channel (Figure 1D) high-density (HD) EMG electrode array with an inter-electrode distance of 10 mm (OT Bioelettronica, Torino, Italy). The HD array was attached to the skin surface at the middle of olecranon process and the styloid process with a double-sided sticker. Prior to the array placement, the skin was scrubbed with abrasive alcohol pads and then cleaned with regular alcohol pads. The EMG signals were sampled at 2,048 Hz with a gain of 1,000 and filtered with a cutoff frequency at 10–900 Hz via EMG-USB2 ^+^ (OT Bioelettronica, Torino, Italy).

### Data Analysis

The neural-drive-based and EMG-based estimates were evaluated using a 500 ms moving window with an overlap of 400 ms between two adjacent windows. Different window parameters tended to influence both estimation approaches in a similar manner. A previous study (10) has demonstrated that the muscle activation of individual finger was localized to particular regions of the muscle. Similar results were further verified in the current study as shown in Figure 1. EMG signals in the channels with low amplitude may contain background noise or components from the co-contraction of other fingers. Therefore, only half of the channels with higher signal amplitude were selected for the decomposition and for the root-mean-square calculation of the EMG. Namely, signals from row 1–8, 5–12, and 9–16 were used for index, ring, and middle fingers, respectively.

#### Electromyogram-Based Estimation

The EMG amplitude has been shown to be the most important and common feature to control external devices. For example, previous studies (11, 12) have indicated that the root-mean-square of EMG exhibited superior performance among the different amplitude-based control features, especially for high-force contractions (≥ 25% MVC). Raw EMG signals were first filtered with a high-pass filter (4th order Butterworth with a cutoff frequency of 50 Hz) to reduce the influence of motion artifacts. A notch filter (2nd order IIR filter at 60 Hz with a bandwidth of 1 Hz) was used to reject the power-line interference. For each sliding window, the root-mean-square of each EMG channel was calculated and then was averaged across all channels as the force estimation. The average of the root-mean-square of HD EMG signals is defined as [1]:

$$\bar{\text{RMS}}=\frac{1}{M}\sum_{i=1}^{M}\sqrt{\frac{1}{N}\sum_{n=1}^{N}x_{i}^{2}\left(n\right)},$$

where *x_i_*(*n*) is the *i*th channel, *n* is the index of sample, *N* is the length of the EMG recording in samples, *M* is the number of EMG channels, and *i* is the index of EMG channel.

#### Neural-Drive-Based Estimation

Raw EMG signals were decomposed into individual MU discharge events using the FastICA method (13) that has been verified as an accurate decomposition algorithm by previous studies (14). All the details of the decomposition algorithm and the parameter selection have been described in Ref. (13, 14). After EMG decomposition, the discharge timings of each individual MU were obtained. The general extension step for EMG decomposition and limitations of the FastICA algorithm (e.g., repeatedly converge to the same MU) usually result in a decomposition output with replicas of the same MU. The replicated MUs were removed for further analysis. Then, the discharge timings of all unique MUs were pooled into a composite spike event train. The composite discharge rate was calculated by dividing the number of events within each window by the window length (500 ms).

The performance of the two approaches was evaluated by the root-mean-square error (RMSE) and the correlation coefficient between the actual force and the estimates. To reduce the interference from the residual muscle activation even when the subject was instructed to relax, the estimation values were subtracted from the baseline values of the initial 2 s of each trial. Due to potential neural-mechanical delay between the neural drive/EMG and the force, the lag cross-correlation coefficient was used to make up the delay before the RMSE and correlation calculations. The lag cross-correlation coefficient was used to find the time delay when the force and the estimate had the highest cross-correlation coefficient. Since the units of EMG (volts), neural drive (Hz) and force (N) are different, the values were all normalized by its maximum value before comparison, leading to a maximum value of 1 (7). The performance differences of the two approaches were compared statistically using paired *t*-tests.

## Results

**Figure 2:**
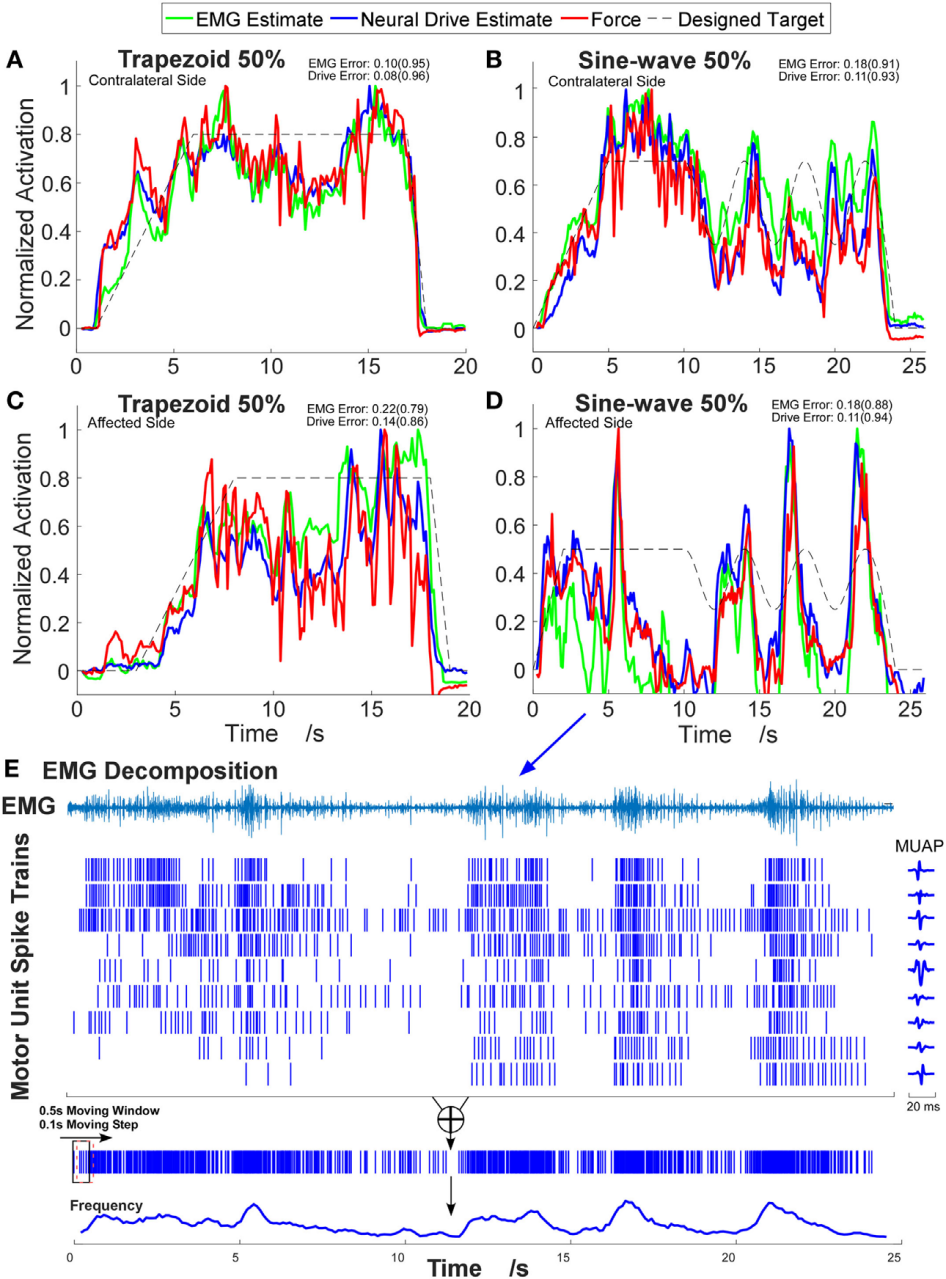
Example time-series plots of four different contraction tasks. **(A,B)** Contralateral side. **(C,D)** Affected side. The corresponding root-mean-square errors (RMSEs) are presented and the correlation coefficients are shown in brackets. **(E)** Illustration of the decomposition results and the neural-drive-based estimation from trial **(D)**. One channel electromyogram (EMG) signal with highest root-mean-square value is shown, and the corresponding waveforms of motor unit action potentials (MUAPs) in that channel are plotted.

A total of 48 trials were analyzed. The number of MUs (mean ± SD) obtained from the decomposition was 7.94 ± 3.60 on the affected side and 10.17 ± 3.17 on the contralateral side. Figure 1 shows the 2-D muscle activation map for index finger from both affected and contralateral sides under the condition of a steady contraction level at 50% MVC. The contralateral side revealed more concentrated and stronger muscle activation, compared with the affected side. The time-series plots of the two force estimation approaches and the corresponding actual forces for the two tracking tasks are shown in Figure 2. In general, the neural-drive-based estimate (in blue) showed a better approximation to the actual force (in red), compared with EMG-based estimate (in green). In addition, the overall results of the RMSE and correlation coefficient are summarized in Table 1. For most cases, the neural-drive-based approach is better than the EMG-based estimation.

**Table 1:** Overall results of the root-mean-square error (RMSE) and correlation coefficient.

		Index		Middle		Ring	
		EMG	Drive	EMG	Drive	EMG	Drive
Sine 20	*C*	*0.15 (0.84)*	*0.22 (0.70)*	0.18 (0.81)	0.14 (0.90)	0.19 (0.86)	0.13 (0.91)
	*A*	*0.22 (0.70)*	*0.27 (0.67)*	0.23 (0.77)	0.19 (0.85)	0.15 (0.90)	0.10 (0.94)
Sine 50	*C*	0.27 (0.81)	0.21 (0.80)	0.19 (0.80)	0.17 (0.83)	0.16 (0.91)	0.10 (0.92)
	*A*	0.18 (0.73)	0.10 (0.94)	0.23 (0.76)	0.19 (0.84)	0.25 (0.75)	0.19 (0.85)
Trapezoid 20	*C*	*0.17 (0.91)*	*0.20 (0.89)*	0.22 (0.84)	0.21 (0.88)	0.16 (0.92)	0.16 (0.92)
	*A*	0.32 (0.32)	0.25 (0.76)	0.14 (0.88)	0.13 (0.89)	0.25 (0.77)	0.15 (0.85)
Trapezoid 50	*C*	*0.10 (0.95)*	*0.16 (0.92)*	*0.12 (0.92)*	*0.13 (0.92)*	0.10 (0.94)	0.10 (0.95)
	*A*	0.33 (0.28)	0.12 (0.95)	0.25 (0.82)	0.22 (0.86)	0.16 (0.93)	0.12 (0.95)

Mean		0.22 ± 0.08 (0.60 ± 0.36)	0.19 ± 0.06 (0.83 ± 0.11)	0.20 ± 0.05 (0.82 ± 0.06)	0.17 ± 0.04 (0.87 ± 0.03)	0.18 ± 0.05 (0.87 ± 0.07)	0.13 ± 0.03 (0.91 ± 0.04)

*The correlation coefficients are written in brackets. C and A represent contralateral and affected sides. Values in italics show the cases that the EMG-based estimation is better than the neural-drive-based estimation*.

**Figure 3:**
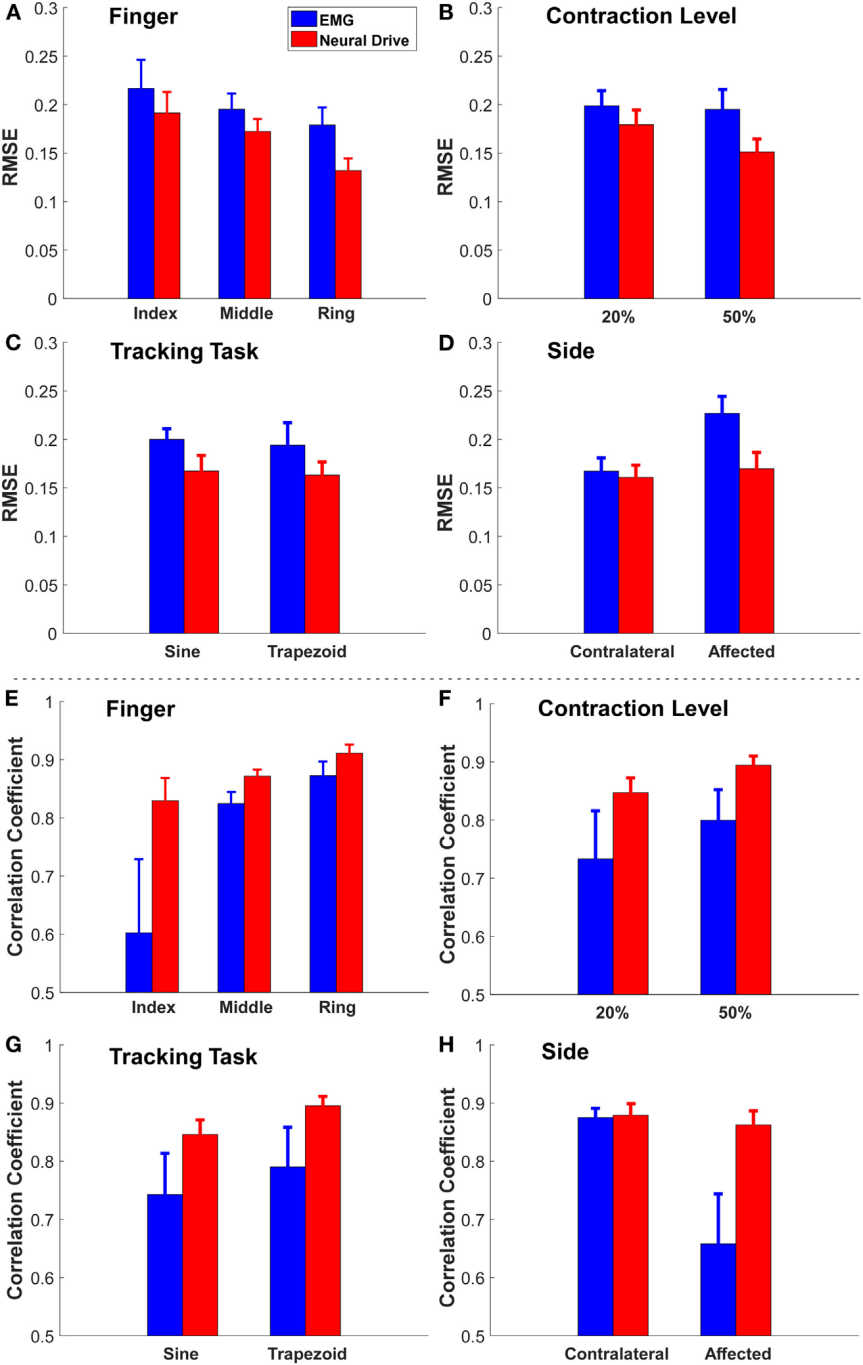
The grand mean ± SE value of RMSE and correlation coefficient of each factor [**(A,E)** finger, **(B,F)** contraction level, **(C,G)** tracking task, and **(D,H)** side]. The error bars represent the SE.

Since four factors (three *fingers* × two *contraction levels* × two *tracking tasks* × two *sides*) were tested, the mean ± SE of the RMSE for each factor was obtained by averaging the values across all the trials among other factors (see Figure 3). Overall, the neural-drive-based estimates showed a significant lower error than the EMG-based estimates [paired *t*-test: *t*(47) = 3.473, *p* = 0.001]. We also observed similar trend for individual factors. Finally, the affected side also showed a larger RMSE than the contralateral side for the EMG-based estimation. Similarly, the neural-drive-based estimates showed a higher correlation with the actual forces than the EMG-based estimates [paired *t*-test: *t*(47) = 3.421, *p* = 0.001].

## Discussion

In this study, we investigated the feasibility of a novel neural drive estimation of the individual finger force of a stroke survivor, based on motoneuron discharge timings at the population level. The discharge timings of the motoneurons can directly reflect the high-level control from the brain. It provides an alternative control input for the simultaneous and proportional control of individual finger forces. In general, our results show that the neural-drive-based approach was superior to the classic EMG-based approach for a majority of the conditions, especially on the affected side.

Since the EMG signal is regarded as a random Gaussian process at the macro level and the information of high-level neural control can be corrupted in EMG signals. The EMG signal varies due to the variation and cancelation of the waveforms of MUAPs at the macro level. Inevitable, external factors, involving ambient background noise, electrode shift, and changes in electrode-skin contact, can also modify the signal properties. These drawbacks can limit the development of a robust and accurate robotic control interface for stroke rehabilitation. In contrast, the neural-drive-based approach intuitively has a better translation of the high level neural control, because the population probability/frequency of the discharge at the MU pool level directly encodes the descending input, without the various limitations in the EMG-based approach. Therefore, neural drive estimate reveals an improvement in the estimation accuracy of the muscle force. Previous studies have shown that the neural-drive-based approach can provide accurate estimation of motor output in different populations such as amputees following targeted muscle reinnervation and intact subjects (7). Our results on the stroke subject also showed consistently better performance in the neural-drive based approach. However, we still found some conditions (in *italics* in Table 1) showing that the EMG-based estimation is better than the neural-drive-based approach, especially in the index finger. One possibility is that the muscle activation generated from index finger of the stroke survivor is weak. The low signal-to-noise ratio (SNR) can decrease the accuracy of the decomposition (13).

Our preliminary results also showed that different factors (*finger, contraction level, tracking task*, and *side*) can affect the estimation of both approaches (see Figure 3). First (*finger*), the magnitude of sEMG signals from the EDC muscle compartment controlling the index finger was weaker than the other two fingers for this subject. Therefore, the low SNR from the index finger had the highest RMSE. Second (*contraction level*), EMG signals from the 50% contraction level had a higher SNR than the 20% condition, and more MUs can potentially be decomposed, which can lead to a better force estimation. Third (*tracking task*), the sine-wave contraction could induce more variations in the action potential amplitudes, compared with the trapezoid contraction, which could limit the neural-drive-based estimation. Nevertheless, the neural-drive-based estimation still revealed improved performance than the EMG-based estimation. Finally (*side*), the tremor during voluntary contractions on the affected side can cause more errors in EMG-based estimation than the contralateral side. In contrast, the neural-drive-based estimation showed similar performance across the two sides.

Our current study only tested a single stroke survivor. Although the results are promising, further testing involving a large subject cohort with different degrees of impairment is clearly needed. In addition, we did not test larger force levels above 50% MVC, because this particular subject had difficulty generating forces continuously at higher levels. During the experiment, the subject may inevitably perform co-contractions, especially during her ring finger extension. The EMG generated from co-contractions can potentially influence the estimation errors in both approaches. Our study was also limited to an offline analysis. Further studies on real-time decomposition are needed before the neural drive control method can be used for real-time applications. In general, our current work shows that the neural-drive-based estimation performed better than the EMG-based estimation for stroke survivors, especially on the affected side. This case study could provide a promising control input for robotics devices during stroke rehabilitation.

## Ethics Statement

This study was carried out in accordance with the recommendations of the local Institutional Review Board (IRB) with written informed consent from the subject. The subject gave written informed consent in accordance with the Declaration of Helsinki. The protocol was approved by the local IRB. The written informed consent was obtained from the participant for the publication of this case report.

## Author Contributions

XH and CD conceived, designed, and performed experiments; CD and YZ analyzed data; XH and CD interpreted results; CD prepared figures; XH and CD drafted manuscript; XH, YZ, and CD revised manuscript; and XH, YZ, and CD approved final version of manuscript and agree to be accountable for all aspects of the work.

## Conflict of Interest Statement

The authors declare that the research was conducted in the absence of any commercial or financial relationships that could be construed as a potential conflict of interest.
